# High prevalence of response to PPI treatment in children and adolescents with eosinophilic esophagitis in southern Brazil

**DOI:** 10.3389/falgy.2024.1346843

**Published:** 2024-04-08

**Authors:** Luiza Salgado Nader, Matias Epifanio, Mariana Guimarães Coelho, Cintia Steinhaus, Melina Melere, Carolina Soares da Silva, Cristina Targa Ferreira

**Affiliations:** ^1^ Division of Pediatric Gastroenterology Unit, Santa Casa de Porto Alegre, Porto Alegre, Brazil; ^2^ Graduate Program in Pediatrics, Federal University of Health Sciences of Porto Alegre, Porto Alegre, Brazil; ^3^ Division of Pathology Unit, Santa Casa de Porto Alegre, Porto Alegre, Brazil

**Keywords:** proton-pump inhibitor responsive esophageal eosinophilia, eosinophilic esophagitis, children, proton-pump inhibitor, histological remission

## Abstract

**Introduction:**

Eosinophilic esophagitis is a newly recognized entity, in which there is significant evidence available that clearly demonstrates the positive impact of PPIs on reducing esophageal eosinophilia in individuals across different age groups, including children, adolescents, and adults. Multiple mechanisms have been proposed to explain how this treatment effect occurs. In Brazil, there seems to be a lack of studies that have prospectively assessed the clinical and therapeutic response rate in pediatric patients with EoE. The objective of this study was to prospectively evaluate the clinical and therapeutic response of pediatric patients with EoE in a medical center located in southern Brazil, by investigating the effectiveness of PPI treatment.

**Methods:**

This study is a clinical, prospective, open trial that took place in a pediatric hospital located in southern Brazil. The focus of the study was on patients diagnosed with Eosinophilic Esophagitis (EoE) who were given treatment using omeprazole/esomeprazole at a dosage of 1 mg.kg per dose, twice daily, for a period of 8–12 weeks. Following the treatment period, the patients underwent another endoscopy. Patients who exhibited 15 or less eosinophils in the biopsy conducted after the treatment were considered as responders.

**Results:**

A total of 27 patients was evaluated (74.1% boys). The average age (± standard deviation) was 8 years (±4). Nineteen patients (70.3%) were considered as responders to PPI treatment: 6 patients—22.2%—exhibited a complete response (defined as having 5 or fewer eosinophil per high power field. Additionally, 13 patients—48.1%—demonstrated a partial response, characterized by eosinophil counts exceeding 5 but less than 15 eos/hpf. When comparing the responder and non-responder groups at presentation, a statistical difference was observed in the prevalence of food refusal as a presenting symptom. Food refusal was found to be more prevalent in the non-responder group (87.5% vs. 26.3%, *P* = 0.008). No differences were observed in terms of atopy history and endoscopic scores. Upon comparing the histological findings from the post-treatment endoscopy of the two groups, it was observed that PPI responders exhibited a greater tendency to decrease basal cell hyperplasia (*P* = 0.06) and intercellular edema (*P* = 0.08).

**Conclusion:**

In this group of pediatric patients with EoE in Southern Brazil most patients showed a high prevalence of histological, endoscopic, and clinical response to PPI treatment. PPIs showed efficacy in Brazilian patients with EoE, most of whom would probably not be able to adequately undergo other treatments.

**Clinical Trial Registration:**

https://ensaiosclinicos.gov.br/rg/RBR-2ntbth9, identifier (U1111-1301-1842).

## Introduction

Eosinophilic esophagitis (EoE) is a chronic, immune-mediated disease with a type 2 inflammatory response. It is characterized by symptoms related to esophageal dysfunction and histologically by the presence of eosinophilic infiltrate in the esophagus, defined as having more than 15 eosinophils per high-power field (eos/hpf) on esophageal biopsy (1).

To establish a diagnosis of EoE, it is necessary to have symptoms indicating esophageal dysfunction and the presence of at least 15 eos/phf on esophageal biopsy. Additionally, it is necessary to rule out non-EoE disorders that could cause or contribute to esophageal eosinophilia (1).

Currently, the prevalence of the disease has been increasing worldwide (2, 3). In Brazil, this number varies from 10 to 50/100.000 people, based on adult and pediatric population (4). The incidence is constantly rising and is estimated to be 10/100.000 cases per year in the country (5). This increase is probably due to a real increase in the disease and, also, due to the result of a greater awareness of it (3).

However, there is no current treatment approved in Brazil that directly disrupts disease pathogenesis.

Standard treatments for EoE include proton pump inhibitors (PPIs), food elimination diets, swallowed topical corticosteroids and esophageal dilations in case of stenosing disease (6). Recent studies show a histological response rate of 1 food elimination diet and 6 food elimination diets of 34% and 40%, respectively (7, 8). Topical steroids had a histological response rate of 59% (7). Nevertheless, these therapies have limitations, and, in clinical practice, food elimination diets have the issue of lower quality of life because of the dietary restrictions and there are concerns about using topical preparations, especially in young children who are using other topical preparations for co-morbid atopic conditions (9).

The use of PPI monotherapy may demonstrate clinical benefits for certain patients. PPIs have a potential to be an effective, primary therapeutic option. Due to their longstanding safety profile and easy administration, patients may choose to initiate treatment with PPIs prior to considering corticosteroids or exclusion diets (10).

The exact mechanism by which PPIs reduce eosinophilia in EoE is still a matter of debate. The proposed mechanisms are gastric acid suppression, which leads to a restoration of esophageal barrier function, and anti-inflammatory effects unrelated to gastric acid suppression. The antisecretory mechanism hypothesis is that the integrity of the esophageal epithelium would be compromised by exposure to gastric acid, leading to the entry of antigens and the activation of an immune response. The hypotheses of anti-inflammatory mechanisms are: (1) PPIs inhibit the migration of inflammatory cells to the esophageal epithelium, blocking the expression of cell surface adhesion molecules; (2) PPIs block STAT6-mediated eotaxin-3 expression, reducing the recruitment of eosinophils to the esophageal epithelium; (3) PPIs can stimulate the aryl hydrocarbon receptor, which normalizes the expression of genes involved in barrier function through inhibition of the IL-4/IL-13- STAT6 pathway; (4) PPIs can inhibit H+-K+-ATPase, which plays a role in eotaxin-3 expression, blocking eosinophil recruitment (11, 12).

We consider the present study relevant and important in clinical practice for populations with difficulties and social disparities, making it difficult to follow diets, and in countries where there are no approved corticosteroids. PPIs become a more inclusive option, as they are less expensive and more feasible being an applicable treatment for this population.

PPIs, despite having a non-optimal response, can help the group of less severe patients, especially children, as these patients will go through different stages in their development. Due to the absence of an approved formulation of swallowed topical corticosteroids in Brazil, the safety profile and convenient administration of PPIs make them an attractive and preferred initial treatment option (10, 13).

In general there is a lack of studies that have prospectively evaluated the clinical and therapeutic response rate in pediatric patients with EoE. This is the first Brazilian study that prospectively evaluates the response to PPIs in pediatric patients with EoE. According to the 2018 AGREE EoE guideline, published studies on the effectiveness of PPIs in treating EoE in children have variable results, probably due to the different doses used and duration of treatment, in addition to different environmental and phenotypic characteristics (1).

The objective of this study was to prospectively evaluate the clinical and therapeutic response of pediatric patients with EoE in a medical center located in southern Brazil, by investigating the effectiveness of PPI treatment. The secondary objective of this study is to assess the associations between demographic factors, clinical characteristics, endoscopic findings and histological features with the response to PPIs.

## Patients and methods

This study is a clinical, prospective, open trial carried out in a Pediatric Hospital located in southern Brazil, from May 2021 to May 2023. Children ranging from 1 month to 18 years of age who were referred to the pediatric endoscopy unit were included. Patients were included when they met the diagnostic criteria for EoE (presenting at least one symptom indicating esophageal dysfunction and the presence of at least 15 eos/hpf on esophageal biopsy) and absence of gastric and duodenal eosinophilia. Four to six biopsies were randomly obtained from the distal and middle esophagus. In order to rule out gastritis and eosinophilic gastroenteritis, biopsy specimens were also collected from the antrum and the duodenum.

Biopsy specimens were stained with hematoxylin and eosin, always analyzed by the same pathologist (M.G.C).

Patients received treatment with either omeprazole or esomeprazole magnesium at a dosage of 1 mg.kg per dose, max dose 40 mg twice a day, for a period of 8–12 weeks. Following the treatment period, the patients underwent another endoscopy. A questionnaire was given to patients to assess symptoms after the first and follow-up endoscopy.

Patients who had 15 or fewer eosinophils in the biopsy obtained after the PPI treatment were considered as responders. A complete response was considered as having 5 or less eos/hpf in all esophageal biopsies taken during the follow-up endoscopy. A partial response was considered to be one with a value of more than 5 and less than 15 eos/hpf. And a nonresponse to PPI therapy was determined by the presence of 15 or more eos/hpf in any of the esophageal biopsies obtained during the follow-up endoscopy.

Children who had previously or recently (up to 1 month before the endoscopy) received treatment with corticosteroids, an elimination diet or PPIs were excluded from the study. In addition, those diagnosed with gastric or duodenal eosinophilia or fungal, viral or caustic esophagitis were also excluded.

Endoscopic findings were described based on the EoE Endoscopic Reference Score (EREFS) established by Hirano et al. (14).

## Statistical analysis

The description of qualitative variables was done by expressing them as absolute (*N*) and relative frequencies (%). When the distributions of quantitative variables were significantly close to normality, they were described using the mean and standard deviation.

The normality of the data was assessed using the Sapiro-Wilk test. Fisher's exact test, on the other hand, was used to test the relationship among qualitative (categorical) variables. The McNemar test was applied for the paired analysis of qualitative variables.

The student's *T* Test for independent samples was employed to test quantitative variables with a normal distribution. A level of statistical significance was considered when *p* ≤ 0.05. The analysis was carried out using the Python programming language in the Google Collab environment. As support, some calculations were performed in SPSS version 23 for Mac (IBM).

Both the patients and their parents consented to take part in the study, which was approved by the ethics committee (number 44070821.9.0000.5683) and the Research Committee of the Hospital da Criança Santo Antônio (HCSA), which belongs to the Irmandade Santa Casa de Misericórdia de Porto Alegre (ISCMPA). A Brazilian clinical trial registry was obtained (number RBR-2nbth9).

## Results

In this study, a sample of 27 patients, ranging from 1 month to 18 years, was diagnosed with EoE and all of them received PPI as first choice treatment.

**Figure 1:**
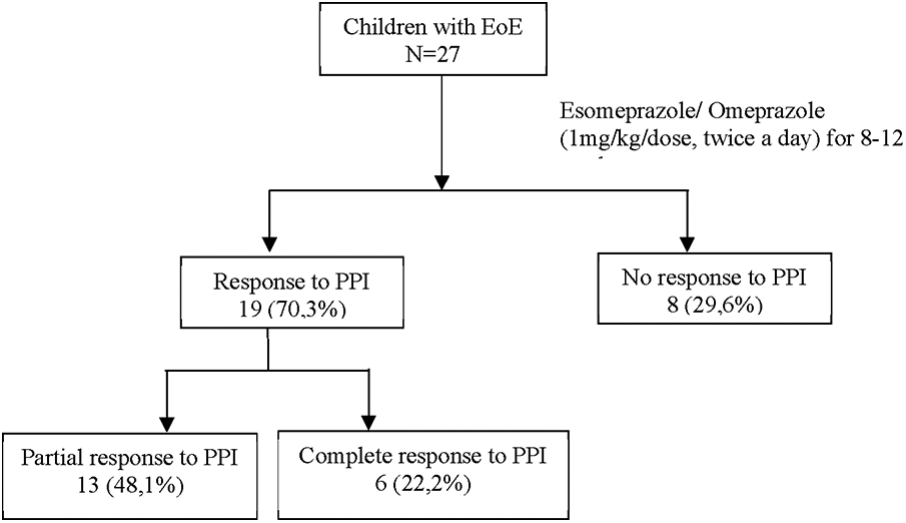
Patient flow. EoE, Eosinophilic Esophagitis; mg, milligram; kg, kilogram; PPI, proton-pump inhibitor.

**Figure 2:**
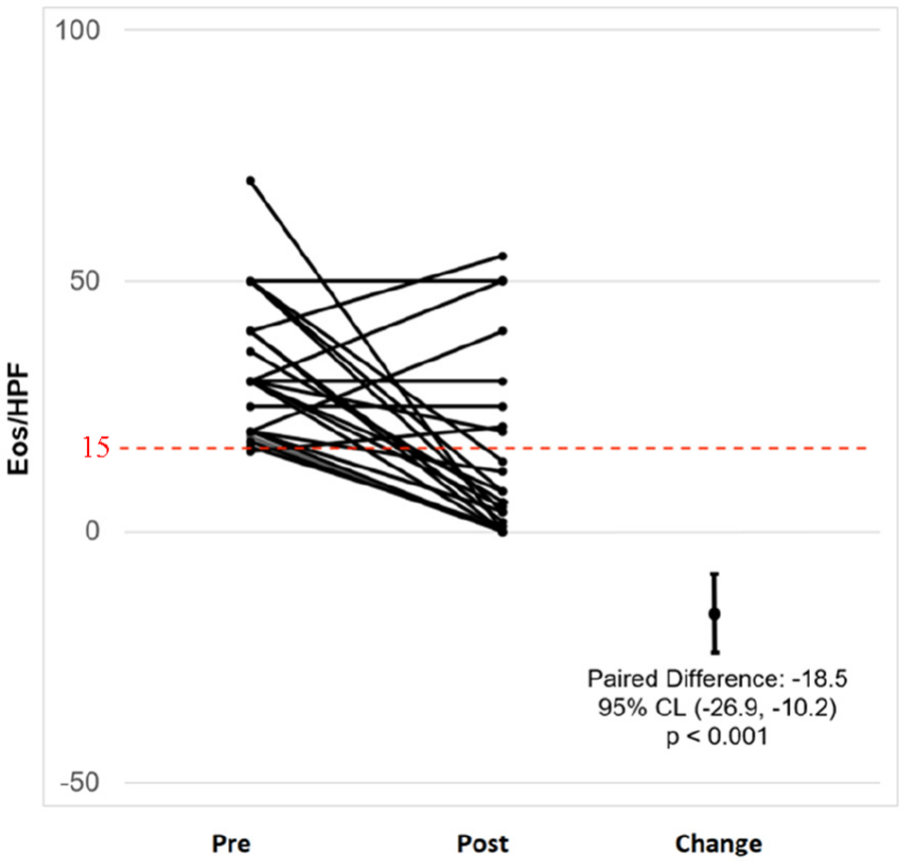
Paired graph with the number of eos/HPF of all individual patients before and after PPI treatment.

19 patients (70.3%) were considered as responders to PPI. 6 patients (22.2%) had a complete response, and 13 (48.1%) had partial response (Figures 1, 2).

Table 1 summarizes the demographic and clinical features of all the responders and non-responders PPI groups.

**Table 1:** Demographic and clinical features of patients with EoE.

** **	All patients (*n* = 27)	PPI responders (*n* = 19)	PPI non responders (*n* = 8)	*p*
Age at diagnosis, *y* (mean ± SD)	8 ± 4	7 ± 4	10 ± 3	0.121
Male (%)	20 (74.1%)	14 (73.7%)	6 (75%)	1.000
White	26 (96.3%)	19 (100%)	7 (87.5%)	0.296
Presenting symptoms	–	–	–	–
- Vomiting/regurgitation	14 (51.9%)	10 (52.6%)	4 (50%)	1.000
- Dysphagia	13 (48.1%)	8 (42.1%)	5 (62.5%)	0.420
- Food impaction	9 (33.3%)	5 (26.3%)	4 (50%)	0.375
- Food refusal	13 (48.1%)	9 (47.4%)	4 (50%)	1.000
- Heartburn	7 (25.9%)	3 (15.8%)	4 (50%)	0.145
- Abdominal pain	14 (51.9%)	9 (47.4%)	5 (62.5%)	0.678
- Chest pain	7 (25.9%)	5 (26.3%)	2 (25%)	1.000
- Feeding difficulties	12 (44.4%)	5 (26.3%)	7 (87.5%)	0.008*
Personal history	–	–	–	–
- Food Allergy	4 (14.8%)	2 (10.5%)	2 (25%)	0.558
- Asthma	10 (37%)	8 (42.1%)	2 (25%)	0.666
- Atopic dermatitis	11 (40.7%)	8 (42.1%)	3 (37.5%)	1.000
- Rhinoconjuntivitis	14 (51.9%)	9 (47.4%)	5 (62.5%)	0.678
Family history	–	–	–	–
- GERD	6 (22.2%)	4 (21.1%)	2 (25%)	1.000
- Asthma	10 (37%)	5 (26.3%)	5 (62.5%)	0.102
- Atopic dermatitis	1 (3.7%)	0 (0%)	1 (12.5%)	0.296
- Rhinoconjuntivitis	16 (59.3%)	11 (57.9%)	5 (62.5%)	1.000

*p < 0.05: statistically significant.

Regarding clinical outcomes, most of the patients (26 children and 96%) reported symptom improvement with PPI treatment after follow-up endoscopy (Table 2). Symptoms were similar in both responders and non-responders to PPIs. Feeding difficulty as an initial symptom was a statistically significant feature in the responder group (*n* = 7/8) (*p* = 0.008).

**Table 2:** Clinical outcome with EoE patients after PPI treatment.

** **	Responders (*n* = 19)	Non responders (*n* = 8)	*p*
Remission of symptoms	18 (94.7%)	8 (100%)	1.000
Partial remission	9 (47.4%)	4 (50%)	0.804
Total remission	9 (47.4%)	4 (50%)	0.804
No change in symptoms	1 (5.3%)	0 (0%)	1.000

Before the second endoscopy all patients were questioned about adherence to treatment and 100% of them reported having adhered to treatment with PPIs.

Pre- and post- treatment endoscopic and histological features are summarized in Table 3. Regarding pre- and post-treatment histological findings, the peak esophageal eosinophil count dropped significantly from the PPI responder group. Furthermore, we observed that the reduction in esophageal eosinophil counts was correlated with an improvement in other histological markers, such as basal cell hyperplasia (*p* = 0.003), edema (*p* = 0.008) in the PPI responder group (Table 3). The non-responder group had a persistence of basal cell hyperplasia and it could be a marker of failure of treatment response (*p* < 0.001) (Table 4).

**Table 3:** Endoscopic and histologic features.

	PPI responders (*n* = 19)			PPI non responders (*n* = 8)		
	EGD1	EGD2	*P*	EGD1	EGD2	*P*
Endoscopic features						
Fixed rings	1 (5.3%)	2 (10.5%)	1.000	3 (37.5%)	2 (25%)	1.000
Exudates	8 (42.1%)	4 (21.1%)	0.219	5 (62.5%)	4 (50%)	1.000
Edema	6 (31.6%)	3 (15.8%)	0.375	2 (25%)	1 (12.5%)	1.000
Furrows	12 (70.6%)	9 (52.9%)	0.375	5 (62.5%)	5 (62.5%)	1.000
Stenosis	0 (0%)	0 (0%)	N/A	0 (0%)	0 (0%)	N/A
Crêpe Paper esophagus	4 (21.1%)	1 (5.3%)	0.250	0 (0%)	0 (0%)	N/A
Ulcers or erosions	3 (15.8%)	2 (10.5%)	1.000	0 (0%)	0 (0%)	N/A
Histological features						
Basal cell hyperplasia	13 (68.4%)	2 (10.5%)	0.003*	7 (87.5%)	7 (87.5%)	1.000
Intercellular edema	10 (52.6%)	2 (10.5%)	0.008*	5 (62.5%)	5 (62.5%)	1.000
Eosinophil abscess	6 (31.6%)	1 (5.3%)	0.125	6 (75%)	4 (50%)	0.625
Fibrosis	0 (0%)	1 (5.3%)	1.000	0 (0%)	0 (0%)	N/A
Eosinophil (hpf)	33 ± 14	4 ± 4	<0.001*	30 ± 11	36 ± 14	0.150

EGD, Esophagogastroduodenoscopy; PPI, proton-pump inhibitor.

*p < 0.05: statistically significant.

**Table 4:** Basal cell hyperplasia in the second endoscopy and treatment response.

** **	Respondedors (*n* = 19)	Non-respondedors (*n* = 8)	*p*
Without basal cell hyperplasia	17 (89.5%)	1 (12.5%)	<0.001*
With basal cell hyperplasia	2 (10.5%)	7 (87.5%)	

*p < 0.05: statistically significant.

## Discussion

Twenty-four patients (47%) had complete remission and 11 (21.6%) had a partial response. The same authors published a cross-sectional multicenter study in 2023, which analyzed the response to PPIs of 387 EoE patients, based on the analysis of the RENESE registry. In this group, 51.4% of patients presented a histological response, 36.9% being a complete response (15). Gómez-Torrijos et al. (16), in a 34 children prospective study in 2018, found a histological response of 26.5%. Lucendo et al. (17), in a systematic review in 2016, which included 188 children, showed a histological response of 54% and a clinical response of 65%, although heterogeneity was high (18).

In this group of pediatric patients with EoE in Southern Brazil, there was a high response rate to PPI treatment. Considering the 4 pediatric prospective studies in the literature (Table 5), our results are similar to Gutiérrez et al. (2) in 2016. The different responses in different locations may reflect variations in severity, doses administered or genetic, phenotype and/or environmental factors.

**Table 5:** PPI efficacy in children: histologic remission (<15 eos/hpf). Studies published in children.

First author and year	Study design	Total number	PPI type	Dose	Duration	Histologic response (%)	Clinical response (%)
Sayej/2009	Retrospective	36	Lanzoprazole, Esomaprazole or Omeprazole	2 mg/kg/day	12 weeks	38.9%	77.7%
Dranove/2009	Retrospective	43	Not reported	Not reported	Not reported	40%	86%
Schroeder/2013	Retrospective	35	Not reported	1–2 mg/kg/day	8 weeks	23%	23%
Rea et al./2013	Prospective	25	Not reported	Not reported	8 weeks	60%	Not reported
Lucendo/2016	Systematic Review	619 (188 children and 431 adults)	Variable	Varying doses according to each study	Variable duration according to each study	50.5%	60.8%
Gutiérrez-Junqueira/2016	Prospective	51	Esomeprazole	2 mg/kg/day, BID	8 weeks	68.8%	82.3%
Gómez-Torrijos/2018	Prospective	34	Omeprazole	2 mg/kg/day, BID	8 weeks	26.5%	Not reported
Harris/2018	Retrospective	64	Not reported	High doses	Not reported	41%	Not reported
Vieira/2020	Retrospective	231	Omeprazole, Pantoprazole or Esomeprazole	2 mg/kg/day, BID	8 weeks	27.7%	Not reported
Rosen/2021	Retrospective	94	Not reported	2 mg/kg/day, BID	Not reported	43.7%	55%
Gutiérrez-Junqueira/2023	Prospective	387	Omeprazole, Esomeprazole or Lanzoprazole	1–2 mg/kg/day, BID	8–12 weeks	51.4%	87.9%
Nader et al./2023	Prospective	27	Esomeprazole or Omeprazole	2 mg/kg/day, BID	8–12 weeks	70.3%	96%

Refererences: Sayet, 2009 (16), Dranove, 2009 (17), Schroeder, 2013 (26), Rea, 2013, Lucendo, 2016 (9), Gutiérrez Junqueira, 2016 (10), Gómez-Torrijos, 2018 (12), Harris, 2018 (27), Vieira, 2020 (28), Rosen, 2021 (29), Gutiérrez Junqueira, 2023 (11). PPI, proton pump inhibitor; Dose, kg, kilogram; mg, milligram; BID, two times a day.

Expanding our understanding of the long-term prognosis for patients with EoE who respond to PPI is crucial (13). These aspects have been investigated in a few pediatric and adult studies. According to two prospective studies, a majority of EoE patients who exhibit a positive response to initial high-dose PPI therapy tend to sustain this response even when the dosage is subsequently reduced (16, 19). Gutierrez-Junquera et al. demonstrated in a pediatric population up to 70% clinical and histological remission in 1 year of follow-up with half the initial dose (1 mg/kg/day).

Histologically, the improvement of basal cell hyperplasia after PPI treatment in responding patients may be an important finding, as there is some evidence that shows the persistence of this histological finding as a predictor of poor response to treatment (20, 21). Clinical improvement was observed in 94.1% of the children treated with PPIs, regardless of whether they achieved histological remission of eosinophils, as also observed in other pediatric studies (2, 15, 22, 23). Gutierréz et al. (2) showed 82.3% clinical improvement despite histological improvement. This study emphasizes that clinical improvement may not reflect a histological improvement, highlighting the importance of performing a endoscopy with esophageal biopsies to assess the response to PPI treatment.

A group of 630 patients, 76 of whom were children, showed a reduction in initial symptoms in 71%. The only statistically significant relationship was between PPIs non-responders and eating difficulties (*p* = 0.018) (24).

Our findings are similar to other pediatric (2) and adult studies that also found no relationship between clinical and endoscopic factors that could predict response to PPIs (25–28).

It is important to consider the limitations and strengths of this study. The biggest limitation of this study was the number of patients, as it was carried out during the COVID-19 pandemic. Other limitations to consider are the rarity of the disease and the even rarer patients who were not treated. Many patients were already receiving some form of OES treatment when the first endoscopy was performed and could not be included in the study.

The strength of this study is that it was a prospective study of new cases of OES in pediatric patients who had not undergone any type of treatment. In addition, the biopsy samples were analyzed by the same experienced pathologist and the same microscope that could have the same lighting configuration, object magnification levels, ocular lens system and image quality.

In conclusion, in this group of pediatric patients with OES in southern Brazil, the majority of patients has a high prevalence of histological, endoscopic and clinical response to treatment with PPIs.

## Data Availability

The original contributions presented in the study are included in the article/Supplementary Material, further inquiries can be directed to the corresponding author.
